# The Two-State Prehensile Tail of the Antibacterial Toxin Colicin N

**DOI:** 10.1016/j.bpj.2017.08.030

**Published:** 2017-10-17

**Authors:** Christopher L. Johnson, Alexandra S. Solovyova, Olli Hecht, Colin Macdonald, Helen Waller, J. Günter Grossmann, Geoffrey R. Moore, Jeremy H. Lakey

**Affiliations:** 1 Centre for Bacterial Cell Biology, Institute for Cell and Molecular Biosciences, The Medical School, Newcastle University, Newcastle upon Tyne, United Kingdom; 2 Centre for Structural and Molecular Biology, School of Chemistry, University of East Anglia, Norwich, United Kingdom; 3 Institute of Integrative Biology, Structural and Chemical Biology, Liverpool, United Kingdom

## Abstract

Intrinsically disordered regions within proteins are critical elements in many biomolecular interactions and signaling pathways. Antibacterial toxins of the colicin family, which could provide new antibiotic functions against resistant bacteria, contain disordered N-terminal translocation domains (T-domains) that are essential for receptor binding and the penetration of the *Escherichia coli* outer membrane. Here we investigate the conformational behavior of the T-domain of colicin N (ColN-T) to understand why such domains are widespread in toxins that target Gram-negative bacteria. Like some other intrinsically disordered proteins in the solution state of the protein, ColN-T shows dual recognition, initially interacting with other domains of the same colicin N molecule and later, during cell killing, binding to two different receptors, OmpF and TolA, in the target bacterium. ColN-T is invisible in the high-resolution x-ray model and yet accounts for 90 of the toxin’s 387 amino acid residues. To reveal its solution structure that underlies such a dynamic and complex system, we carried out mutagenic, biochemical, hydrodynamic and structural studies using analytical ultracentrifugation, NMR, and small-angle x-ray scattering on full-length ColN and its fragments. The structure was accurately modeled from small-angle x-ray scattering data by treating ColN as a flexible system, namely by the ensemble optimization method, which enables a distribution of conformations to be included in the final model. The results reveal, to our knowledge, for the first time the dynamic structure of a colicin T-domain. ColN-T is in dynamic equilibrium between a compact form, showing specific self-recognition and resistance to proteolysis, and an extended form, which most likely allows for effective receptor binding.

## Introduction

Intrinsically disordered proteins (IDPs) have emerged in the last decade as a challenging and exciting area of structural biology (1). Generally inaccessible to x-ray crystallography, their functions often include molecular recognition of structured targets whereby binding is linked to folding of the IDP (2). However, in many cases, the bound form does not have a single conformation or has extensive unfolded linker regions leading to fuzzy complexes (3). Furthermore, one IDP may bind to several different folded targets and vice versa, thus creating interaction hubs where intermolecular competition also occurs (4). In this way, IDPs continue to force reevaluation of our understanding of molecular interactions in both eukaryotic and prokaryotic cell biology. Elucidation of structure-function relationships in IDP is rarely achieved by a single structural method and combinations of complementary techniques give the best insights into their structure and interactions. One important characteristic is the measure of the IDP’s elongation, compared with globular proteins, expressed as the hydrodynamic radius or Stokes radius (1), or as the radius of gyration, *R*_*g*_ (5), originally derived from fundamental studies of folding states for globular proteins (6, 7). Moreover, recent advances in the interpretation and modeling of data from analytical ultracentrifugation (AUC) and small-angle scattering experiments have made these invaluable tools to measure molecular dimensions in solution (8, 9, 10). Combined with NMR, the classic approach to structural studies of IDP (11), these solution structural techniques give a comprehensive view on the micro- and macrobehavior of IDPs.

In this article, we investigate the native conformation of the antibiotic protein colicin N, which has an N-terminal, 90-residue, intrinsically disordered region (IDR) (1, 12). This is especially interesting because most colicins contain unfolded N-terminal domains. This commonality suggests a generic role for unfolded translocation domains because they share very little sequence homology and interact with many different targets. In the example studied here, the colicin N IDR interacts with multiple protein targets OmpF and TolA, which are also targeted by multiple IDPs ColE9, ColA, ColE1, TolB, etc. (13, 14). As a result, these colicins are good examples of interaction hubs using IDP.

Gram-negative bacteriocins are large, >40 kDa, water-soluble, antibacterial proteins. Produced by bacteria such as *Escherichia coli* colicins (13), *Pectobacterium* (15), and *Yersinia pestis* pesticins (16), they must cross the Gram-negative outer membrane to kill competing bacteria. This requires the presence of specific outer membrane and periplasmic protein receptors (13) that act together to form translocons. However, the exact means by which colicins overcome this barrier is unknown and, if understood, could reveal a bacterial Achilles’ heel that could be targeted by antibiotic developers.

**Figure 1:**
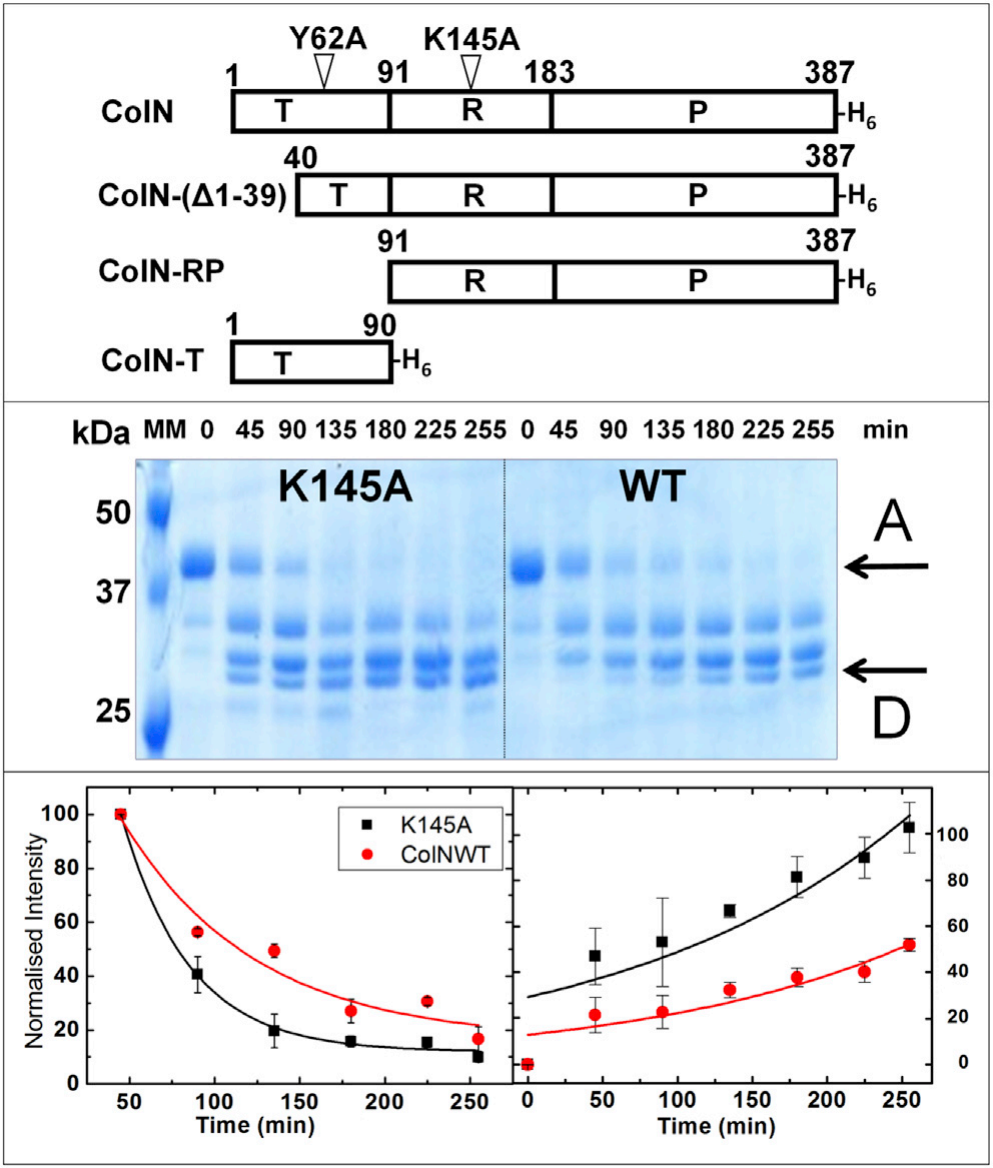
K145A mutation increases T-domain disorder. (*Top panel*) Shown here are Colicin N constructs and mutants used in this study. T, translocation domain; R, receptor binding domain; P, pore forming domain; H6, hexahistidine tag. Numbers correspond to amino acid residues. K145A and Y62A are separate mutants. (*Middle panel*) Limited proteolysis by trypsin was analyzed by SDS-PAGE. Four bands A–D are visible; A corresponds to intact ColN_1–387_ and D the shortest fragment ColN_93-387_ in which the T-domain is entirely removed. (*Lower panel*) Right side shows decreasing band density of Band A. Left side shows increasing band density of Band D. Both show faster proteolysis kinetics for K145A (*filled squares*) compared with WT (*filled circles*). The similar increased proteolytic sensitivity caused by the mutation Y62A has been published previously (23). To see this figure in color, go online.

Colicins comprise three functional domains—an N-terminal translocation (T-) domain that usually contains an IDR, a central receptor binding (R-) domain, and a C-terminal toxic domain (13) (Fig. 1). However, the structure of pectocin, an atypical bacteriocin without a T-domain IDR, has recently been reported (15).

We study colicin N (ColN), which is the smallest pore-forming colicin at 42 kDa, and which kills the target cell by inserting its C-terminal pore-forming (P)-domain into the cytoplasmic membrane. The receptor binding R-domain of colicin N binds to lipopolysaccharide (LPS), the main lipid component of the outer surface of *E. coli* (17). All 90 N-terminal residues of the T-domain IDR are missing from the published x-ray structure PDB: 1A87 (18), but are of interest because they define the binding sites for its other receptors—the outer membrane protein OmpF ColN residues 1–15 (19, 20) and the periplasmic protein TolA ColN residues 40–67 (12, 14, 21, 22). These are termed the “OmpF Binding Site” (OBS) and “TolA Binding Site” (TABS), respectively.

NMR has revealed much about the structure and behavior of ColN-T (21, 23, 24), including the dual recognition function of the T-domain, in which the TABS also binds to a site on colicin N itself. Furthermore, in the ColN TABS mutant Y62A, which does not bind TolA, self-recognition of ColN-RP is also abrogated and NMR showed the T-domain to be more disordered and protease-sensitive. This 90-residue IDR thus has two bound states—a pretoxic state held in a less dynamic form and which is resistant to proteolysis, and an active state bound to TolA and OmpF when the target cell is found (23). In other words, the flexible T-domain of colicin N is chaperoned until membrane insertion, whereupon it is enabled to carry out at least two separate binding events. This has been termed “regulated unfolding” (25). Here we seek to define the dynamic native state of colicin N T-domain and understand how the transformation into an active toxin takes place.

## Materials and Methods

### Protein purification

ColN_1–387aa_ (ColN), ColN_40–387_ (ColN-Δ1-39), ColN_90–387_ (ColN-RP), ColN_1–90_ (ColN-T), and mutants ColN-Y62A and ColN-K145A each had a –SSHHHHHH at their C-terminus and were purified as previously described (20, 26), followed by dialysis into 50 mM sodium phosphate, pH 7.6, 300 mM NaCl. For HSQC, NMR ^15^N-labeled proteins were obtained from bacteria grown in M9 minimal medium containing 1 g/L ^15^NH_4_Cl as the only nitrogen source. Limited proteolysis using trypsin (from Sigma-Aldrich, St. Louis, MO) was performed as previously described (23). The measurement of ANS binding using a barycentric mean analysis was as described previously for nonheated samples (27).

*NMR spectroscopy*. NMR spectra were acquired with a Unity Inova (Varian, Cary, NC) or Avance III spectrometer (Bruker, Billerica, MA) equipped with triple resonance and pulsed field gradient probes, operating at ^1^H frequencies of 499.865, and 800.229 MHz, respectively, and ^15^N frequencies of 50.66 and 81.09 MHz, respectively, at 15°C. Resonance assignments of ^13^C/^15^N-labeled R domain were obtained from standard triple-resonance experiments, and assignments of ColN and ColN-T were taken from the literature (23). Spectra were processed using the software NMRPipe (28) and analyzed in the software NMRView (29). Before Fourier transformation, a cosine-bell window function was applied to each dimension for apodization. The indirect dimensions were first linear-predicted to double the number of data points, and then zero-filled to round up the number of data points to the nearest power of two. ^1^H chemical shifts were referenced directly to external two, 2-dimethyl-2-silapentane-5-sulfonate sodium salt and the ^13^C and ^15^N chemical shifts indirectly to 2-dimethyl-2-silapentane-5-sulfonate sodium salt (30).

*Analytical ultracentrifugation*. Sedimentation velocity experiments were carried out in a ProteomeLab XL-I Analytical Ultracentrifuge (Beckman Coulter, Palo Alto, CA) using both absorbance at 280 nm and interference optics. All AUC runs were carried out at a rotation speed of 48,000 RPM at 4°C, using an eight-hole An-Ti50 rotor and double-sector aluminum-Epon centerpieces (Beckman Coulter, Brea, CA). The sample volume was 400 *μ*L and the concentrations were ranged between 0.3 and 1.5 mg/mL and usually up to seven sample dilutions were used. The partial specific volumes $\bar{v}$ for the proteins were calculated from the protein amino acid sequence, using the program SEDNTERP (31) and extrapolated to the experimental temperature as in (32). The density and viscosity of the buffer 20 mM Tris, 150 mM NaCl, pH 7.5 at the experimental temperature was also calculated using SEDNTERP. Sedimentation velocity profiles were treated using the size-distribution *c*(*s*) model implemented in the program SEDFIT (33). To determine the mass of each species, the c(s) distribution was converted to a *c*(*M*) distribution. Each peak on the distribution plot was integrated to obtain the weight-averaged values for the sedimentation coefficient and molecular mass. The integrated values of sedimentation coefficient *s* obtained at experimental conditions were converted to the standard conditions *s*_20,w_, which is the value of the sedimentation coefficient in water at 20°C and extrapolated to zero concentrations of protein.

*Hydrodynamic parameters and folding state*. The theoretical Stokes radii *R*_*s*_ for native state *N*, molten globule *MG*, premolten globule *PMG*, intrinsically disordered coil, and denatured urea/guanidinium chloride proteins were calculated as described in (6) using the value of monomeric molecular mass *M* calculated from the amino acid sequence:

(1) $$\begin{array}{l} \mathrm{Log}\left(R_{S}^{N}\right)=-\left(0.204\pm 0.023\right)+\left(0.357\pm 0.005\right)\times \mathrm{Log}\left(M\right)\\ \mathrm{Log}\left(R_{S}^{\text{MG}}\right)=-\left(0.053\pm 0.094\right)+\left(0.334\pm 0.021\right)\times \mathrm{Log}\left(M\right)\\ \mathrm{Log}\left(R_{S}^{\text{PMG}}\right)=-\left(0.21\pm 0.18\right)+\left(0.392\pm 0.041\right)\times \mathrm{Log}\left(M\right)\\ \mathrm{Log}\left(R_{S}^{D\left(\mathrm{urea}\right)}\right)=-\left(0.649\pm 0.016\right)+\left(0.521\pm 0.004\right)\times \mathrm{Log}\left(M\right)\\ \mathrm{Log}\left(R_{S}^{D\left(\mathrm{GdmCl}\right)}\right)=-\left(0.723\pm 0.033\right)+\left(0.543\pm 0.007\right)\times \mathrm{Log}\left(M\right)\\ \mathrm{Log}\left(R_{S}^{ID\left(\mathrm{coil}\right)}\right)=-\left(0.0551\pm 0.032\right)+\left(0.493\pm 0.008\right)\times \mathrm{Log}\left(M\right)\\ \mathrm{Log}\left(R_{S}^{ID\left(\mathrm{PMG}\right)}\right)=-\left(0.239\pm 0.055\right)+\left(0.403\pm 0.012\right)\times \mathrm{Log}\left(M\right) \end{array}$$

and used to estimate the theoretical sedimentation coefficients including typical hydration term as follows:

(2) $$\begin{array}{l} f=6\pi \eta R_{s},\\ s=\frac{M\left(1-\bar{v}\rho \right)}{N_{A}f}{\left(\frac{\bar{v}}{\bar{v}+\delta_{1}{\bar{v}}_{1}^{0}}\right)}^{1/3}, \end{array}$$

where *f* is the frictional coefficient of the molecule; *η* is the solvent viscosity; *ρ* is solvent density; $\bar{v}$ and ${\bar{v}}_{1}^{0}$ stand for the partial specific volumes of protein and solvent, respectively; *δ* is a hydration *g*_water_/*g*_protein_; and *N*_*A*_ is the Avogadro’s number. The expressions in Eq. 2 are applied to infinitely dilute solutions with no interparticle interactions.

### Small-angle x-ray scattering

*Data collection*. Small-angle x-ray scattering (SAXS) data for colicin N samples were collected on the Beamline BM29 (34) at the European Synchrotron Radiation Facility in Grenoble, France. Scattering curves were recorded at a wavelength of 1.008 Å at a sample-detector distance of 2.85 m covering the momentum transfer range 0.05 < *q* < 0.45 Å^−1^, where *q* = 4*π*sin*θ*/*λ* and 2*θ* is the scattering angle. Sample concentrations used ranged between 7 and 0.5 mg/mL. Usually five sample concentrations were measured and the experimental temperature was 4°C. Initial data processing and averaging were carried out according to (35). Data were corrected for buffer scattering and scaled for concentration using the software PRIMUS (36). The data were checked for radiation damage and concentration-induced aggregation.

The experimental SAXS data were submitted to the SASBDB database (https://www.sasbdb.org/) as entries SASDC23 (ColN WT), SASDC33 (ColN Δ1-39), SASDC43 (ColN K145), and SASDC53 (ColN-T).

*Flexible domain modeling*. The flexible translocation domain was modeled using both the program BUNCH (37) and the ensemble optimization method (EOM) (38). BUNCH was used to model the full-length ColN molecule, comprising the flexible part of the translocation domain and the rigid part R and P domains (PDB: 1A87). The model was fitted simultaneously against two experimental data sets of ColN, the full-length protein, and its truncation mutant, ColN-Δ1-39.

The distributions of *R*_*g*_ and *D*_max_ for plausible protein conformations were modeled using the EOM package (38), employing a two-step procedure: 1) random chain generation using the program RANCH, and 2) then selection of the most repeated conformations generated by the program GAJOE, on the basis of a genetic algorithm. A pool of 20,000 nativelike or random-coil structures based on the amino acid sequences of ColN-WT, ColN-T, and ColN-Δ1-39 were generated in the first step of modeling using the tools implemented in the program RANCH. To model ColN-WT and ColN-Δ1-39, the high-resolution structure of the folded RP domain was included (PDB: 1A87). To model ColN K145A, the atomic coordinates of lysine 145 in PDB: 1A87 were changed to alanine using the program COOT (39, 40). In the second stage, ∼15 conformations were selected in 50 cycles of the genetic algorithm. The final model consisted of the pool of conformations of polypeptide chains found to fit to the experimental scattering curve, minimizing the discrepancy between the experimental *I*_exp_(*q*) and calculated *I*(*q*) curves,

(3) $$\chi^{2}=\frac{1}{K-1}{\sum_{j=1}^{K}\left[\frac{\mu I\left(q_{j}\right)-I_{\exp}\left(q_{j}\right)}{\sigma \left(q_{j}\right)}\right]}^{2},$$

where *K* is the number of experimental points, *σq* values are standard deviations, and *μ* is a scaling factor.

*Hydrodynamic modeling*. The program HYDROPRO (41) was used to calculate the hydrodynamic parameters of the structures modeled from the SAXS data. The atomic element radius (AER) for the dummy atom models was chosen as 3.5 Å whereas for EOM models AER was chosen as 4.5 Å. The choice of AERs was based on the estimation of increase in particle volume that resulted from the hydration layer generated by the program. We chose the value for AER so that the amount of bound water would be 0.3–0.4 *g*_water_/*g*_protein_ (42).

Alternatively, the EOM-selected structures were optimized using procedures implemented in COOT (39, 40) (i.e., the initial EOM models were refined with correct population of the residue side chains and followed by an energy minimization procedure implemented in the same program). Their hydrodynamic parameters were calculated using the program SoMo (43, 44, 45) as a more suitable hydrodynamic tool for studying disordered proteins, which could have different hydration compared with globular folded proteins (6).

*Calculation of R*_*g*_ *for random walk polymer/polypeptide*. The expected *R*_*g*_ for disordered polypeptide was calculated with the following equation (46):

(4) $${\left(R_{g}\right)}^{2}=b^{2}\left[\frac{y}{6}-\frac{1}{4}-\frac{1}{4y}+\frac{1}{8y^{2}}\right],$$

where *y* = *L*/*b*. The chain contour length *L* can be expressed as *L* = *n* × *a* × *f*, where *n* denotes the number of amino acid residues in polypeptide; *a* = 3.78 Å is the characteristic dimension of one residue; and *f* is the geometrical factor equal to 0.95, which takes into account the constraints of polypeptide chain (46). The *b* value is twice the persistence length and expresses the rigidity of the polypeptide chain. The *b* value was assumed to be 20 Å for a completely disordered random walk polymer, based on the published literature (47). For the 98-residue construct ColN-T(1-90)-SSHHHHHH, this gave a calculated *R*_*g*_ of 355 Å.

## Results

### K145A disrupts the native complex of the T-domain

The *β*-sheet region of the ColN-R domain displays a concave face, and we thought this could be a binding site for LPS. We created the mutation K145A to remove a centrally exposed cationic residue in an attempt to inhibit both receptor binding and toxicity. This failed, as its activity against *E. coli* was unchanged. However, it was observed that, upon storage, the T-domain degraded more quickly than usual. Limited proteolysis assays confirmed this by showing that the T-domain of ColN-K145A was more sensitive than the WT to proteolysis by trypsin (Fig. 1). The degradation rate is similar to, but slower than, that of ColN-Y62A, a TABS mutant within the T-domain that inhibits both TolA binding and self-recognition (23). The interesting aspect of ColN-K145A is that it is not within the disordered T-domain itself but on the surface of the folded R-domain, which binds to LPS (17) in the initial stages of ColN import. It is also the first indication we have of where ColN-T may interact with the rest of ColN. We also investigated the folded state of the two mutants Y62A and K145A using a microvolume ANS binding assay (27), which showed greater exposure of hydrophobic regions in the mutants compared with the wild-type (WT) protein (Fig. S1 *A*). Far UV-CD difference spectra compared with WT of the two mutants showed a larger change, with more disorder for ColN-Y62A than ColN-K145A (Fig. S1 *B*). Thus, three independent measurements indicate that the T-domain is more disordered in the mutants, and two indicate that ColN-Y62A is more unfolded than ColN-K145A.

### K145A and Y62A affect different regions of the T-domain

**Figure 2:**
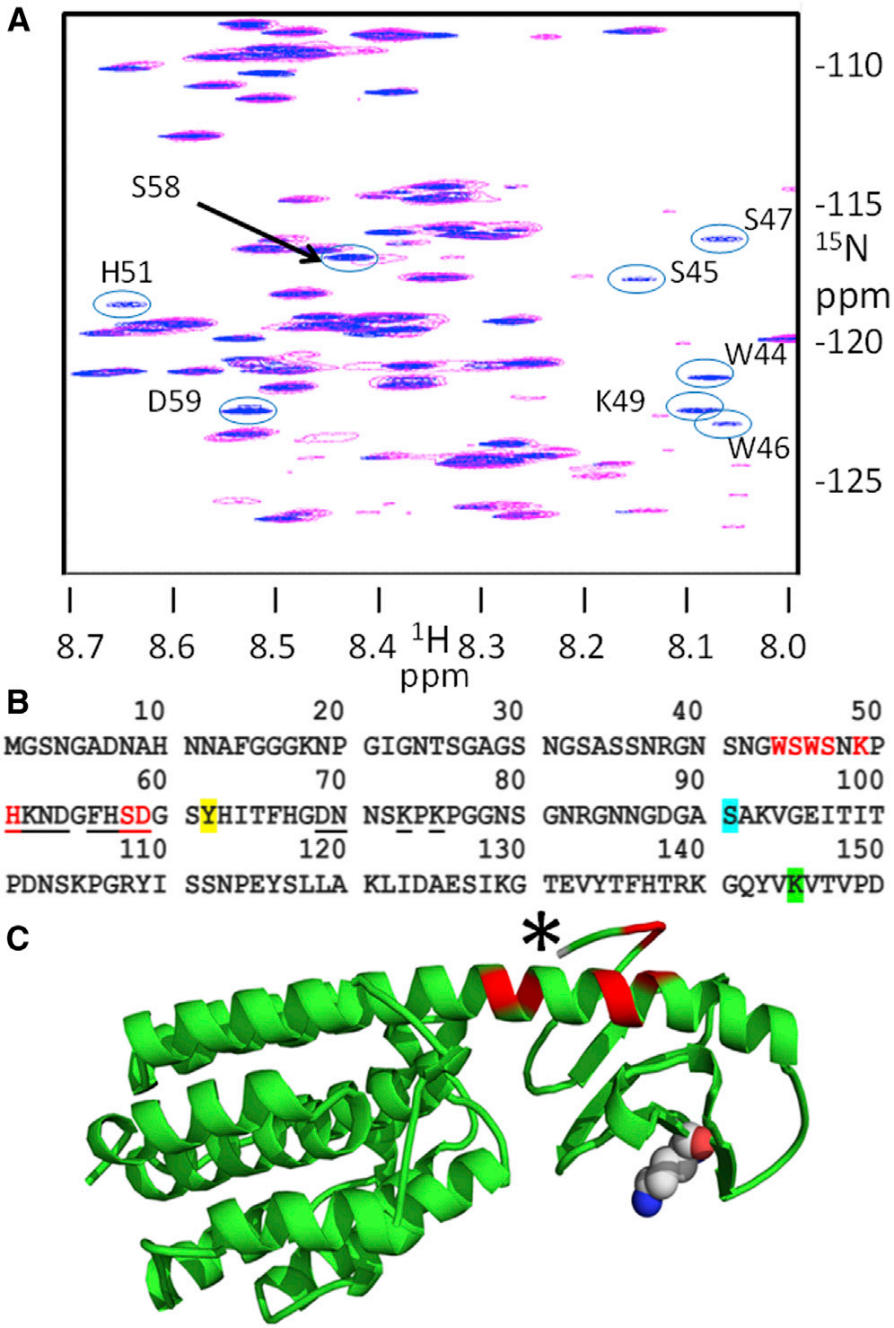
NMR defines interacting regions. (*A*) HSQC-NMR spectrum of ColN-WT (*pink*) and ColN-K145A (*blue circled*) and labeled (*blue*) peaks in the mutant spectrum become visible due to reduced self-recognition and increased mobility of that residue in the mutant. (*B*) Given here is the primary structure of residues 1–150 of ColN. Y62 is highlighted in yellow and K145 in green. The first residue visible in the x-ray structure S91 is in cyan. Residues in mutants that display HSQC-NMR peaks only in the mutant spectra and not in the WT are emphasized; ColN-K145A is in red (see (*A*) above) and ColN-Y62A is underlined (23). (*C*) Given here is the x-ray crystallographic structure of Colicin N residues 91–387 starting at the asterisk, showing K145 in space-filling style and interaction sites revealed in the HSQC-NMR spectrum of ^15^N labeled ColN-RP mixed with free ColN-T in red (24).

In aqueous solution, free ColN-T (residues 1–90) has a well-resolved ^15^N-^1^H HSQC NMR spectrum in which the majority of backbone amide proton resonance peaks can be assigned to specific residues. In full-length colicin N, self-recognition and thus decreased mobility of ColN-T residues leads to the disappearance of a number of ColN-T-specific peaks belonging to residues 40–67 within the TABS (23, 24). In the ^15^N-^1^H HSQC spectrum, the mutation ColN-Y62A caused 12 T-domain peaks to reappear, which revealed the release of specific residues normally involved in self-recognition (23). The residues affected ranged from H51 to K75, which surround the Y62A mutation. A similar result was observed here for K145A (Fig. 2 *A*), but this is structurally more revealing because the changes in the ColN-T spectrum result from a mutation outside of ColN-T. The seven mobilized residues in ColN-K145A correspond to the start of the TABS between W44 and D59 (Fig. 2 *B*). Thus, the region of ColN-T that is mobilized by the ColN-K145A mutation 44–59 is N-terminal to that mobilized by ColN-Y62A 51–75. Also, the effect of K145A appears spatially more limited than that of Y62A. In a previously published experiment, addition of free unlabeled ColN-T to ^15^N-labeled ColN-RP altered the chemical shifts of several amide protons corresponding to a limited number of ColN-RP residues (24). This indicated binding of the isolated T-domain to ColN-RP. The spectrum of ColN-RP has now been partially assigned, and the affected residues map to the regions V94, G95, E96, and I97 and Q179, L181, L182, F183, E187, and E189 (Fig. 2 *C*), which are on the helix and loop next to the first resolved residue S91 in the high-resolution x-ray crystallographic model. These combined results further define the extent of the self-recognition site on the R-domain, and show that K145 may interact with the region that includes residues 40–60.

### The isolated T-domain is in equilibrium between extended and compact conformations

The ColN translocation domain ColN-T, monomeric in solution, has been shown experimentally to be unstructured (12, 48), in agreement with its PONDR analysis (Fig. S2) (49). However, in analytical ultracentrifugation, ColN-T sediments itself as a compact particle with a sedimentation coefficient that is within the range corresponding to a native state or molten globule (see Fig. S3 *A*; Table S1), in agreement with FRET (48) and NMR diffusion (21) studies.

**Figure 3:**
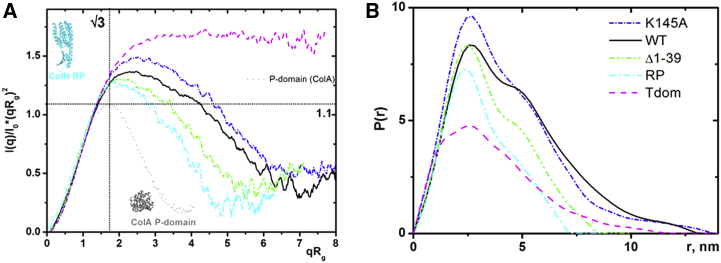
SAXS data demonstrate different levels of disorder in ColN constructs. (*A*) Shown here is an *R*_*g*_-based, dimensionless Kratky plot where *R*_*g*_ is the radius of gyration Å, *q* is the momentum transfer or scattering vector Å^−1^, *I*(*q*) is the scattered intensity at the given value of *q*, and *I*_0_ is the scattering intensity at *q* = 0. The plot shows that the degree of disorder in colicin constructs increases with the length of the T-domain and that the K145A mutant has a higher level of disorder compared with WT. The data from a virtually globular colicin domain ColA-P are shown for comparison; ColN-RP, which lacks the disordered T-domain, is more asymmetric than the isolated P-domain, and this is reflected in the shape of the plot. (*B*) The distance distribution function *P*(*r*) displays elongated shapes in all constructs that contain the T- domain. The same color code/line type is used for each sample on both panels. To see this figure in color, go online.

SAXS curves represented in the form of Kratky plot *q*^*2*^*Iq* versus *q*, where *q* is the scattering angle and *I*(*q*) is the scattering intensity, provide information about the globularity of a particle in solution (50). The dimensionless version of the Kratky plot in which $\left(Iq/I_{0}\right)q^{2}$ versus *q* is converted into $\left(Iq/I_{0}\right){\left(qR_{g}\right)}^{2}$ versus *qR*_*g*_, proposed several years ago (51), gives a useful semiqualitative insight into the conformation of flexible molecules in solution (Fig. 3). The scattering of globally compact particles presented on a Kratky plot will have a distinctive bell-shaped maximum, whereas flexible or/and asymmetric particles will show a deviation from this shape with displacement of the curve maximum; an extreme case will be a completely random polymer chain represented by a saturation-like curve with a plateau at high angle (more details can be found in (10) and http://www.bioisis.net/tutorial/). The dimensionless Kratky plot $\left(Iq/I_{0}\right){\left(qR_{g}\right)}^{2}$ versus *qR*_*g*_ for the isolated T-domain derived from SAXS data resembles a saturation-like curve, suggesting an unstructured molten globule conformation without any residual secondary structure (Fig. 3 *A*). The distance distribution function gives the maximal dimension *D*_max_ of the particle at ∼115 Å (Fig. 3 *B*).

**Figure 4:**
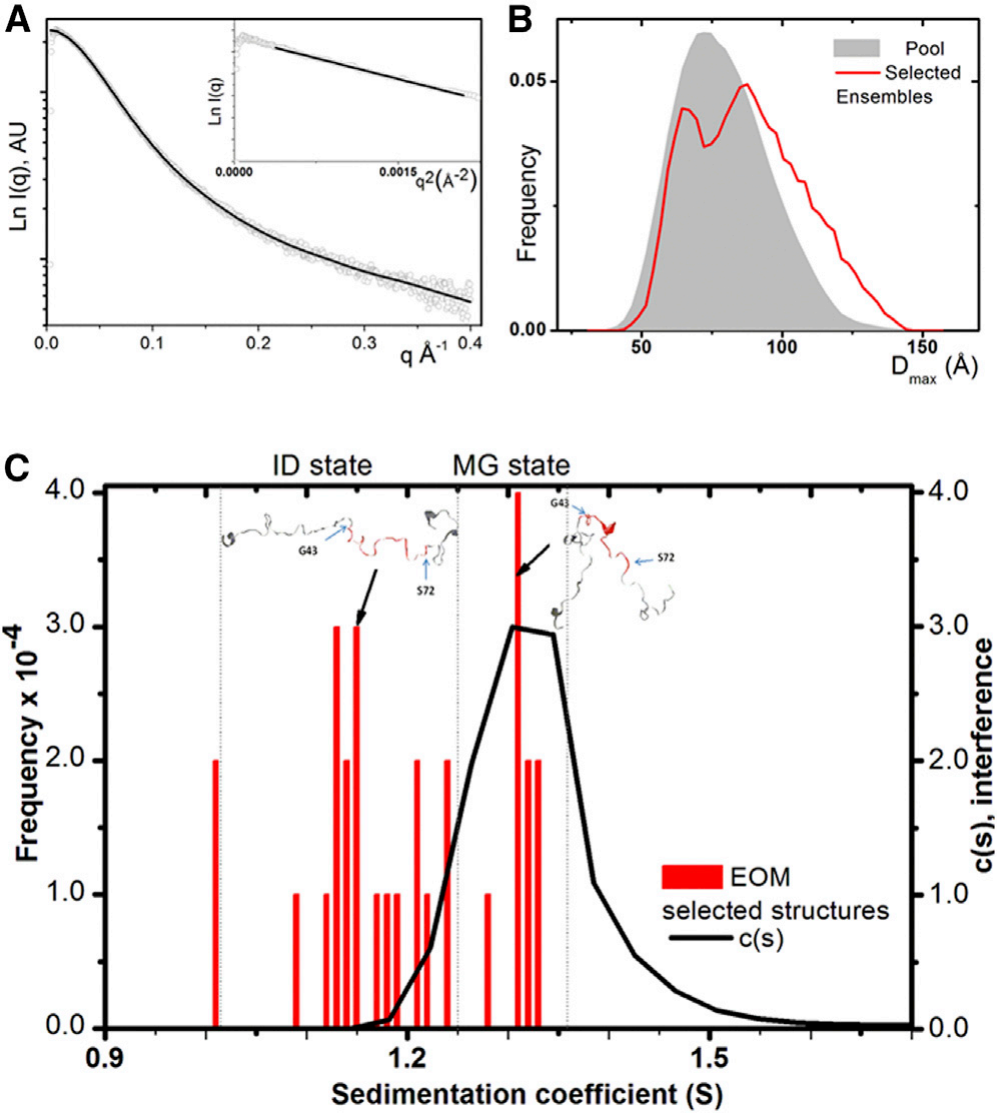
The isolated T-domain can exist simultaneously in both compact molten globule and extended intrinsically disordered states. (*A*) The data, including an inset showing a linear Guinier region, show no signs of aggregation of this isolated, unfolded, domain. (*B*) The size-distribution *D*_max_ of conformational ensembles generated by EOM, fitted to the data in (*A*), reveals two separate peaks in the selected population of conformations versus the general pool. (*C*) The T-domain size-distribution *c*(*s*) obtained from the AUC experiment (*line*) is superimposed with sedimentation coefficients (*s*) calculated for a selected pool of EOM-generated conformations (*bars*). These conformations are considered to be instantaneous and describe the range of conformations from molten globule (*MG*) state to intrinsically disordered (*ID*) state but the extended form’s low values of *s* are not detected by AUC. The examples of EOM-generated models illustrating the ID and MG conformations are depicted with the residues glycine 43 and serine 72 highlighted, which indicate the extremes of the region identified by NMR to be immobilized by self-recognition in full-length WT ColN). The sedimentation coefficient is expressed in Svedberg units (*S*); the AUC *c*(*s*) distribution is represented in arbitrary units derived from the interference dataset. To see this figure in color, go online.

No aggregation was detected on the T-domain scattering curve because the Guinier plot for the T-domain could be approximated satisfactorily by linear dependence, as shown in Fig. 4 *A* (*inset*). SAXS data were treated as a flexible system using the EOM (38, 52). The EOM results suggest the presence of two main populations within the size distribution of the T-domain (Fig. 4 *B*). The fit to the data is shown in Fig. 4 *A* and yields a good agreement between experimental data and curve fit *χ*-value = 1.22 without any systematic deviations. The size-distribution *D*_max_ of structures fitting the ColN-T scattering curve revealed two major peaks centered around 65 and 93 Å, respectively, with the selected population of conformations shifted toward more disordered particles versus the general pool of structures. The distribution of radii of gyration *R*_*g*_ displays a similar shape (Fig. S3 *B*), with the main peaks at 23 and 29 Å. According to Eq. 4 (see Materials and Methods), the statistical length for the first population of ColN-T conformers is 9.5 Å, which corresponds to a chain persistence length of 4.25 Å ∼1.3 residue per length; thus, this conformation can be defined as “quasi-native”; the second population of conformers with *R*_*g*_ values centered around 29 Å is characterized by a statistical length of 15.5 Å, which is 7.8 Å in terms of persistence length, thus corresponding to 2.8 residues per length. The latter population represents an extended disordered polypeptide segment with virtually no compact regions. Thus, the quasi-native ColN-T population relates to compact structures and agrees well with sedimentation velocity results, whereas the second one relates to an extended form, which, surprisingly, was not detected by AUC (Fig. 4 *C*). The occurrence of extended and compact conformations of solution-forms of the T-domain obtained from EOM are shown in Fig. 4 *C*, superimposed upon the *c*(*s*) distribution derived from sedimentation velocity experiments. It should be noted, however, that this comparison could be done only in the constrained pool of EOM-selected structures considered to be instantaneous conformations, which provides defined atomic coordinates from which to calculate sedimentation coefficients. This fact results in significantly lower numbers for population frequency compared with *R*_*g*_ or *D*_max_ histograms obtained directly from scattering data. Two illustrative examples of modeled ColN-T extended and compact conformations are shown in Fig. 4 *C*. Based on the values of hydrodynamic radius and sedimentation coefficient (calculated by using either SoMo (44) or HYDROPRO (41)), these conformations could be classified as instantaneous examples of ID and MG states by applying the expressions in Eqs. 1 and 2 (see Materials and Methods), respectively.

Using NMR diffusion methods, we previously measured the hydrodynamic radius, also called the Stokes radius (*R*_*S*_), for the isolated T-domain to be 19.3 Å (21), which is in agreement with both the theoretically estimated *R*_*s*_ for a molten globule state (19.2 Å) (Table S1) and AUC experiments. The value of *R*_*S*_ obtained from sedimentation velocity experiment was 20.6 ± 1.45 Å. The EOM-derived pool of compact conformations with *R*_*g*_ around 23 Å could be also classified as a quasi-native population by applying the known dependence between the values of *R*_*g*_ and *R*_*S*_ (for globular proteins, the ratio *R*_*g*_/*R*_*S*_ = 0.775, whereas for denatured proteins *R*_*g*_/*R*_*S*_ ≅ 1.4) (6, 53). Referring to the above-mentioned NMR and AUC measurements, the *R*_*g*_/*R*_*S*_ ratio was 1.19 and 1.12, respectively.

### The level of intrinsic disorder in ColN increases with the length of T-domain and is enhanced in ColN K145A

Fitting the SAXS data using ab initio dummy atom models resulted in a reliable fit for ColN-RP residues 91–384, thus confirming the quality of the data collected. However, as the amount of T-domain increased, and especially when ColN-K145A was modeled, the fits became increasingly poor (Fig. S4). From this, it was clear that we needed to use similar approaches in modeling the full-length proteins as we had used for the T-domain.

Using the SAXS data, the dimensionless Kratky plots *qR*_*g*_^2^*Iq/I*_0_ versus *qR*_*g*_ for all the T-domain-containing ColN constructs WT, Δ1-39, and K145A (Fig. 3 *A*) show the results typical for a combination of a folded, compact fragment with a disordered segment tail (54). The least disorder is found in the ColN-Δ1-39 deletion mutant, which has the most compact T-domain conformation, i.e., lacking the N-terminal 39 residues not constrained by self-recognition within the TABS. This showed a similar Kratky plot to the ColN-RP construct, which lacks the T-domain entirely and, interestingly, in the distance distribution *P*(*r*) plots, both ColN-Δ1-39 and ColN-RP are more compact than the much smaller, isolated T-domain, confirming the importance of the self-recognition behavior in constraining ColN-Δ1-39 (Fig. 3 *B*). The data for the purely globular ColA pore-forming domain (friction ratio 1.14), calculated by SoMo, are shown for comparison with ColN-RP (friction ratio 1.22,) demonstrating in a semiquantitative way the increased asymmetry for the ColN-RP molecule and all other ColN constructs (Fig. 3 *B*). The full-length ColN, which includes residues 1–39, shows a more extended particle in solution that is even more pronounced in the ColN-K145A mutant. Aggregation of the Y62A samples, observed by AUC, precluded their analysis by SAXS. The *P*(*r*) plots of ColN and ColN-K145A describe elongated particles (55), which is in good agreement with the results of analytical ultracentrifugation (Figs. 3 *B* and S3 *A*; Table S1). Further information about the methods can be found in the Supporting Material.

### Determining the dynamics of full-length Colicin N by SAXS

Complementary structural studies of full-length molecules and their deletion mutants are a commonly used approach in the analysis of complex molecules containing disordered fragments (52). Because the dimensionless Kratky plot for ColN Δ1-39 showed a compactness level roughly similar to ColN RP, an attempt was made to fit, using CRYSOL (56), the experimental scattering curve for both ColN WT and the Δ1–39 deletion mutant using a structural homology model for the full-length molecule generated by I-TASSER (57) (Fig. S5). Because residues 90–387 have a direct homology with the solved x-ray model, the I-TASSER structures consist of this region with variable T-domain structures. The resulting models position the T-domain TABS region close within 11 Å to the R-domain (residues 95–97).

**Figure 5:**
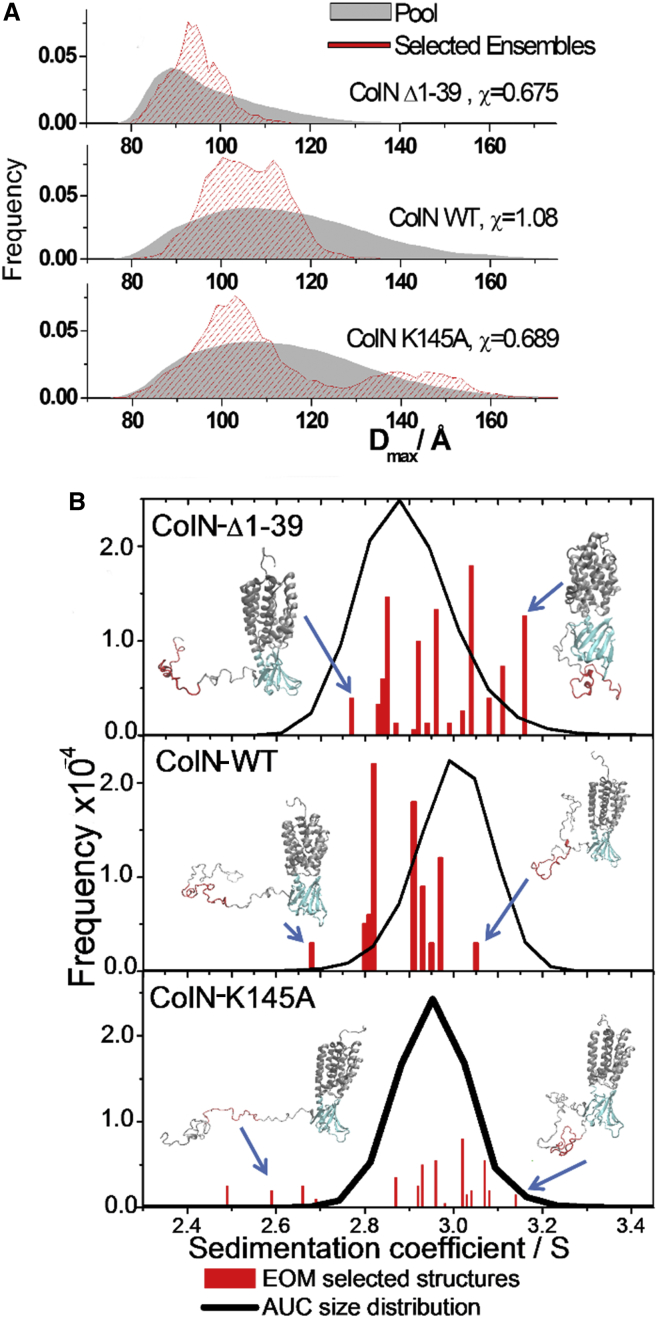
Extended and compact T-domains in full-length ColN. (*A*) The size-distribution *D*_max_ of structures fitted, using EOM, to the scattering data (Fig. S5) reveals that ColN-K145A displays two well-separated peaks in the selected population of conformations versus the pool of generated structures. ColN-WT shows a narrower distribution that nevertheless is broader than ColN-Δ1-39. (*B*) Size-distribution *c*(*s*) value obtained from AUC data analysis (*line*) is superimposed with the calculated sedimentation coefficients of the SAXS-derived, EOM-selected, structures (considered to be instantaneous); the bars describe a range of conformations but the extended forms are not detected by AUC (*line*). The sedimentation coefficient is expressed in Svedbergs (*S*). To see this figure in color, go online.

The ColN-Δ1-39 scattering curve was fitted satisfactorily (*χ* = 0.735) by CRYSOL with a rigid single structure generated by I-TASSER, suggesting a relatively well-defined conformation (Fig. S5 *A*, *bottom panel*), whereas the full-length ColN molecule cannot be fitted in this way (*χ* = 2.09) (Fig. S5 *A*, *top panel*). This supports the NMR data that show residues 1–39 as disordered (23). Furthermore, by introducing the NMR-determined flexibility into the EOM in residues 72–90, the ColN-Δ1-39 mutant data fit improved further the EOM fit (*χ* = 0.673) (Fig. S5, *A* and *B*). This approach was extended to the WT ColN, where each stipulated flexible region 1–39 and 72–90 could be modeled separately (Fig. S5 *C*) and the contribution of the flexible regions can be seen in total conformation space. It is worth noting, however, that in the more general case, when the T-domain region is modeled as being totally flexible, the *R*_*g*_ (Fig. S3) and *D*_max_ (Fig. 5 *A*) distributions occupy the same conformational space as when modeled as a compact TABS region surrounded by flexible fragments (the actual EOM fit of the scattering curves is shown in Fig. S6). Thus, in general, full-length ColN and its truncated mutant, ColN-Δ1-39, can be modeled with EOM without a priori restrictions and characterized by a distribution significantly narrower than that expected for a random pool of 20,000 randomly generated polypeptide chains of the given sequence (see Materials and Methods) (Fig. 5 *A*). The unrestricted fit of *R*_*g*_ to the data is shown in Fig. S5 with *χ*-values of 0.675 and 1.08, respectively, for ColN-Δ1-39 and ColN. The equivalent *D*_max_ distribution generated by EOM for ColN shows a bimodal form with peaks at ∼100 and ∼117 Å (Fig. 5 *A*). We previously estimated from *K*_*D*_ values that the extended form of ColN-WT would represent ∼5% of the population (23), and the EOM modeling (Fig. 5 *A*) agrees with this by showing a minor second peak for the WT protein. However, the EOM fit for ColN-K145A (Fig. S6), with a *χ*-value of 0.689, resulted in a size distribution (Fig. 5 *A*) that clearly defines two distinct, separated peaks—one similar to the compact peak of ColN-WT (*D*_max_ ∼ 100 Å), and a second one representing ∼25% of the total population at ∼145 Å, which represents a significantly more extended and mobile structure. Sedimentation coefficients for selected conformations generated by EOM (considered to be instantaneous) were calculated and are presented in Fig. 5 *B* superimposed with independently obtained *c*(*s*) distribution from sedimentation velocity AUC experiments. Because the TABS is shown by NMR to have reduced mobility, it is of particular interest to compare the configuration of the TABS in the EOM models with the observed hydrodynamic behavior of ColN (Fig. 5 *B*). The calculated sedimentation coefficient of selected ColN-T domain structures taken from the EOM ensemble increases as the TABS becomes more compact by forming a tight loop (*highlighted*) and consequently approaches the experimentally observed AUC value. Similarly, one observes for the full-length construct a compaction of the T-domain taking place when it comes within reach of the R-domain (Fig. 5 *B*). Conversely, in the case of the extended fraction for the K145A mutant, the T-domain is more extended and the TABS is often situated at a considerable distance from the compact R- and P-domains.

## Discussion

This work sought to clarify two aspects of colicin N: 1) how does the self-recognition affect the spatial mobility of the ColN-T, and 2) can we define the inherent flexibility of the colN-T and understand how it accesses its twin targets OmpF and TolA?

### The role of the TABS as conformational instigator within the disordered T-domain

It has been shown that IDP exists within a spectrum of compaction, ranging from structures with hydrodynamic radii close to that of a folded protein to largely extended structures (1). A compact structure is correlated with several features such as increased polypeptide length and hydrophobic residue content, decreased net charge and proline content and, perhaps unexpectedly, histidine tags (58, 59). The latter are relevant because all the proteins studied here contain histidine tags. However, in all cases except for the isolated ColN-T, we added the histidine tag to the folded C-terminal domain, well away from the disordered region and thus assume its effects on the IDR are not significant. In ColN-T, the Ser_2_His_6_-tag follows directly after residue 90, so may induce compaction but, due to ColN-T’s protease sensitivity, we cannot remove it proteolytically. When the hydrodynamic radius *R*_*S*_ is calculated, including the effect of the His-tag, according to (58), a value of 23.5 Å is predicted compared to the 28.9 Å expected for a chemically denatured state (48). However, the value for *R*_*S*_ measured by NMR is 19.3 Å (21). Thus, by either calculation, the T-domain can be considered compact. This global value, however, hides the complex dynamics of the isolated T-domain. Results presented here and elsewhere (14, 23, 24, 60) point to the TABS region, with its aromatic groups, as the driver for compaction. More precisely, NMR chemical shift analysis suggests that residues 62–67 form a *β*-structure in the isolated T-domain (24), which is in harmony with predictions (14). When bound to TolA the TABS is predicted to also form a *β*-hairpin, which extends the existing TolA *β*-sheet (14, 23, 24). Thus, binding of the TABS supports the process of conformational selection. The TABS belongs to the group of IDRs, which are relatively rich in aromatic residues and for which restricted conformations can be experimentally detected in the unbound form (60, 61). The SAXS data for ColN-RP predicts a conformation in solution that agrees with the known x-ray structure and thus provides a benchmark for the SAXS/AUC modeling approach used for the unknown structures of ColN, ColN-Δ1-39, and ColN-K145A. However, the SAXS data analysis for these longer molecules yielded significant discrepancies between the expected and fitted protein volumes (Fig. S4), underlining the need to account for additional structural flexibility. Further support came from inspection of Kratky plots (Fig. 3 *A*), which indicate additional disorder for all constructs that include regions of the T-domain. A combination of rigid body modeling combined with an additional N-terminal flexible polypeptide segment using BUNCH (37) (Fig. S4 *B*) not only led to better fits to the experimental SAXS curves but also predicted sedimentation behavior similar to the observed AUC data (see Table S2). The fits implied that the isolated T-domain is extended in all cases (Table S1). However, due to its flexibility, a single conformation for the T-domain in solution cannot adequately describe the experimental data, and the EOM approach was used to analyze the conformational heterogeneity (52). Starting with the isolated T-domain, this analysis showed a predominantly bimodal distribution of shapes with the maximum distances *D*_max_ of 65 and 93 Å, respectively. Thus, the EOM resolves two populations, compact and extended (Fig. 4), which suggests that the conformational states occupy separate energy minima separated by an activation barrier. It is not clear why the AUC studies only detected a compact structure similar to the smaller of these two, but presumably the reason that previous NMR studies (21) also only detected one structure is that the chemical exchange between the compact and extended structures was rapid enough on the NMR timescale to yield averaged signals weighted by populations, which were heavily in favor of the compact structure. An alternative explanation for unresolved by AUC ColN-T extended conformers’ population could be its susceptibility to hydrostatic pressure effects developing in AUC cells at high rotation speeds, and translating it into the more compact conformation. A similar phenomenon was reported for pharmaceutical antibodies that have flexible regions as part of their structure (62).

### Self-recognition in colicins

When the TABS ColN 40–67 is free to interact with the remainder of colicin N, and particularly the R-domain, it drives self-recognition concomitant with reduced disorder of the IDR, even in the case of a mixture of separate ColN-T and ColN-RP protein domains (23). Self-recognition is a well-known and recurring theme in colicins because none of the IDRs in structurally characterized colicins are completely disordered (60). In full-length ColN, residues 1–39 appear to be fully disordered but a truncated protein ColN-Δ1-39 lacking this region has not yielded suitable crystals for x-ray crystallographic studies. This can be understood from the SAXS data because, although we can obtain a plausible dummy atom model from SAXS for ColN-RP, the fitting results for ColN-Δ1-39 improve significantly when a distribution of flexible conformers, delineated by EOM, are taken into account. The bimodal distribution of EOM structures, found for ColN-T, was also observed for the longer constructs, ColN and most clearly ColN-K145A, but not ColN-Δ1-39. This suggests that the two-state ColN-T behavior is observed whenever the full-length T-domain is present.

ColN-Y62A was initially defined as a mutant which, by suppressing TABS binding to TolA, was not toxic to *E. coli* (12). We then discovered that ColN-Y62A suppressed T-domain self-recognition (23), and here we show that ColN-K145A, which is on the structured receptor binding R-domain, causes similar but less extensive disordering reduction of self-recognition of the T-domain. We previously estimated the WT self-recognition equilibrium constant to be 0.055, such that 95% of the TABS was bound to ColN at any time (23). The smaller fraction of 5% may be represented by the extended structure corresponding to the less pronounced peak at larger *D*_max_ in the distribution plot for ColN (see Fig. 5). The picture becomes much clearer with ColN-K145A, which shows a distinct two-state and more extended T-domain population (Fig. 5), in agreement with biochemical (Fig. 1) and NMR data (Fig. 2). The structures, selected by the EOM analysis that describes the extended species, often contain compact peptide sections, which correlate with the TABS region. To ensure the quality of the samples for solution scattering SAXS studies, we performed AUC sedimentation velocity experiments. K145A sedimented more slowly than WT colicin N, confirming its more disordered state. Unexpectedly, several separately purified samples of Y62A exhibited aggregation and were therefore not suitable for SAXS experiments. The mutation Y62A removes a hydrophobic residue from an IDR, which might justifiably be expected to increase solubility, and thus provides more evidence that, albeit disordered, these segments exist in finely balanced, stable conformations that can be disrupted by single residue substitutions, just like folded globular domains. Fortunately, ColN-K145A, which is less disordered than ColN-Y62A, behaves well in solution and provided clear data in both AUC and SAXS experiments. However, why it inhibits self-recognition by the TABS remains unclear. We initially assumed that the TABS interacted directly with K145 and might allow us to define the self-recognition site on the structured R-domain of colicin N. This appeared to agree with the occurrence near Y62 of three out of the five rare negatively charged residues in ColN-T, implying a possible electrostatic interaction with K145. Furthermore, in all selected EOM modeled structures, the T-domain is found on the opposite side of the R-domain to K145, which is in agreement with the interaction data from NMR (Fig. 2). Because the modeled structures could be influenced by the orientation of the N-terminal end of the R-domain, we tried adding various constraints of fixing Y62A within 8 Å of K145A but the resulting EOM ensembles showed poorer fits to the data, so currently, and surprisingly, we have no evidence for a specific interaction between T-domain and K145A.

### Interactions with partner proteins

The primary functions of the T-domain are to bind outer membrane protein F (OmpF) at the cell surface and TolA within the periplasm. The ColN-T/OmpF interaction (20) employs an OBS at the extreme N-terminus which, by analogy with the well-characterized colicin E9 (63, 64, 65), probably enters the central pore of OmpF. Deletion of the OBS has little effect on toxicity of colicin N in the laboratory (20), but because OmpF is the most efficient Omp for transmembrane translocation (19), the OBS may help guide ColN toward the most productive translocon. Here we show that the OBS within ColN-Δ1-39 is always disordered and available for interaction. The TABS needs to access the TolA protein, which is beyond the outer membrane barrier, and, as in the case of Col-E9, this may occur by threading the OBS and TABS through the OmpF pore (63). This will need to first overcome the self-recognition and our current work indicates how this may be achieved. We show that in solution the T-domain exists in a two-state equilibrium. The compact self-recognition form may protect against proteolytic degradation and enable faster diffusion toward the target. The transiently extended conformations make it possible for the T-domain to thread through OmpF and if, during such an extended phase, it binds to either OmpF or TolA this form will be stabilized. Thus, although conformational selection of preformed epitopes may play a role in the final binding step, the work here simply reports on the ability of the OBS and TABS to reach their receptors via significantly extended conformations. The mutant ColN-K145A shows that the equilibrium can be easily shifted and therefore the binding of LPS by the R-domain could displace TABS into its extended form ready to insert into OmpF. LPS binding is essential for colicin N toxicity and, although the binding site remains to be defined, it is, like self-recognition, largely contained within the R-domain (17). Because the required core sugars on the LPS are likely to be buried in pure LPS regions of the membrane, it is more likely that ColN-R binds to them where they are exposed at the periphery of membrane proteins such as OmpF (17). In such a scenario, the accessible OBS may bind OmpF, whereas the R-domain locates LPS. By overcoming self-recognition, the binding of LPS to ColN may stabilize the extended form of the TABS, enabling it to enter the cell and initiate translocation across the outer membrane (66). Full-length ColN has a strong affinity for LPS, so there is no need for an OmpF binding step to uncover the LPS binding site. Also, OBS shows no evidence of constraint by self-recognition because, in contrast to TABS affinity for TolA, the affinity of OBS for OmpF is similar in ColN-T and full-length ColN (20). Thus, the sequence and interplay of OmpF and LPS binding by ColN remains to be elucidated. In any scenario, it would seem that the dynamics of the T-domain revealed in this article will be beneficial to constrain the T-domain until its receptors are within range. Further experiments on T-domain dynamics in the presence of LPS are needed but will be complicated by the need for micellar LPS in the NMR and SAXS experiments.

### Advantage of combining complementary structural techniques

The results from NMR, AUC, SAXS, and EOM have brought the study of colicin dynamics to a new level. SAXS can obtain reliable data from such proteins in solution, but attempts to resolve the unfolded state clearly indicate the need for a distribution of conformers. However, instead of a poorly defined data set, the clear identification of a two-state equilibrium, assisted by the discovery of K145A, helps us resolve the self-recognition state and understand how it works in vivo. The EOM-modeled structures are independently refined by the genetic algorithm and these consistently represent the TABS as a compact conformation whereas the other T-domain sections show various degrees of disorder. This independently supports the TABS ordering previously identified by mutagenesis, NMR, and PONDR analysis (23, 24). This independence of data underlines the reliability of the modeling approach, even though it may underestimate the level of structure achieved by the TABS region; not all known constraints such as the molecular contacts observed by NMR can be included. Nevertheless, the ensemble of structures generated agrees both generally and locally with experimental observations. Our data thus show that the IDR of colicin N has an inherent flexibility that, on the one hand is restrained by self-recognition, and on the other hand, is in dynamic equilibrium with a second, more extended state, which is likely to increase the efficiency of receptor binding.

## Author Contributions

C.L.J. and H.W. carried out molecular biology and protein biochemistry experiments and protein purification. O.H., C.M., and G.R.M. performed and analyzed NMR experiments. A.S.S. performed and analyzed SAXS and AUC experiments. C.L.J., J.H.L., and J.G.G. contributed to SAXS experiments and data analysis. A.S.S. and J.H.L. wrote the article.

## Supporting Citations

References (67, 68, 69, 70, 71, 72) appear in the Supporting Material.

## Supporting Material

Document S1. Supporting Materials and Methods, Figs. S1–S6, and Tables S1 and S2

Document S2. Article plus Supporting Material
