# Supplementary material for: The Two-State Prehensile Tail of the Antibacterial Toxin Colicin N
Source: Biophys J. 2017 Oct 17;113(8):1673–84. doi: 10.1016/j.bpj.2017.08.030 (PMC5647543; doi:10.1016/j.bpj.2017.08.030)
Supplement: Document S1. Supporting Materials and Methods, Figs. S1–S6, and Tables S1 and S2 [file mmc1.pdf]

Biophysical Journal, Volume *113*

Supplemental Information

The Two-State Prehensile Tail of the Antibacterial Toxin Colicin N

Christopher L. Johnson, Alexandra S. Solovyova, Olli Hecht, Colin Macdonald, Helen Waller, J. Günter Grossmann, Geoffrey R. Moore, and Jeremy H. Lakey

Supporting Material

All T-domain containing ColN constructs are homogeneous asymmetric particles (monomers) in solution

The solution states of the various ColN constructs were studied by sedimentation velocity analytical ultracentrifugation. All samples of ColN, except Y62A, were shown to be monomers represented by a single main peak on a size-distribution plot (Figure S3). Several separate purification batches of ColN Y62A revealed the presence of higher aggregates indicating the unexpected result that this point mutation, which removes a hydrophobic residue in a disordered region, reduces the solubility of this protein. This mutant was thus excluded from further study by AUC. The sedimentation parameters (sedimentation coefficient and mass) for ColN-WT, ColN-Δ1-39, ColN-K145A, ColN-RP (ColNΔ1-90) and ColN-T (ColN1-90) were obtained by fitting the sedimenting boundaries in the program SEDFIT (1]) using the non-interacting discrete species model (rmsd<0.01) and listed in Table S1. The resulting numbers for the mass are in a good agreement with those calculated from the amino acid sequence and the friction ratio suggests a slightly elongated shape for the ColN molecule. ColN-WT and K145A have almost the same mass but the fit for the mutant (f/f_0) shows it to be more extended in good agreement with the proteolysis, ANS, CD and NMR data (Figs. 1 and S1). The sedimentation parameters of ColN-RP which only contains the residues observed in the crystal structure were in good agreement with those calculated from its atomic structure (2) (PDB : 1A87), data presented in Table S1. The ColN-Δ1-39 mutant, which lacks residues 1-39 none of which are involved in self-recognition, sediments as a compact version of ColN-WT.

Modelling approaches to resolve the contribution of the dynamic IDP

Ab initio modelling methods The distance distribution function (P(r)) for each protein was calculated with both GNOM (3) and Bayesian estimation (http://bayesapp.org/) (4,5). In this work we mostly relied on a Bayesian approach in determining P(r) where the noise level and particle's maximum dimension both estimated by the program and probability distributions for the parameters can be calculated. We also used GNOM to determine P(r) on limited occasions to create an input for rigid body modelling. In this case the quality of experimental data fit was the main criteria for chosen D_{max} value, probed firstly with $P(0)\neq 0$ (to estimate whether any constant background present), $P(D_{max}) \neq 0$ (to judge whether the chosen interval is correct), finalising the data treatment with the conditions $P(0)=0$, $P(D_{max})=0$, providing also, that $D_{max}s_{min} < \pi$.

Low-resolution molecular shapes were restored as dummy atom models (DAMs) using DAMMIN (6). DAMs were generated 32 times from single scattering data sets and corresponding P(r) functions used to establish a degree of similarity for the models which were then averaged to find the most common shape. All 30 models were superimposed and averaged using the DAMAVER package (http://www.embl-hamburg.de/ExternalInfo/Research/Sax/damaver.html). Low- and high-resolution structures were superimposed using the program SUBCOMB20 (7).

In the case of the compact ColN-RP the *ab initio* dummy atom model (DAM) was in good agreement with its crystal structure and hydrodynamic data. On the other hand all other ColN envelopes derived *ab-initio* had much larger volumes than would be expected for a protein of the same given mass (Fig S4 (panel A, inserts)). The discrepancy between the anhydrous volume of the particle and dummy atom model (DAM) significantly exceeded the usual difference resulting from a typical protein hydration (0.3-0.4 $g_{water}/g_{protein}$) (Table S1). The excessive fitted volumes of the full length ColN constructs is reflected also in the fact that hydrodynamic parameters (such as sedimentation coefficient) calculated using coordinates of DAMs were significantly under-estimated compared with the s-values obtained in sedimentation velocity experiments (Table S2). However, this discrepancy was eliminated by averaging a large number of DAMs: after averaging 30 models the calculated hydrodynamic parameters appear to be very close to the experimentally measured numbers. Since ColN-RP, which lacks the disordered domain, fits well to its *ab initio* DAM, we believe the excessive volumes of the other models are likely to result from the unstructured translocation domains. Thus, an approach was employed to comprehensively characterise the solution structure of full-length ColN.

We applied a combined rigid body and *ab initio* modelling approach as is available in BUNCH (8) to model the ColN constructs, which comprised all three domains (either partially or in full). The atomic coordinates of receptor and pore-forming domains (PDB code 1A87; (2) were exploited as the rigid body portion of the molecule and the unknown T-domain was modelled as a flexible, unstructured region (the model for ColN-WT is illustrated in Fig.S6B and calculated hydrodynamic parameters of the model are given in Table S1). The resulting models fitted the experimental data neatly with χ-values of 0.82, 1.32 and 0.98 for ColN-Δ1-39, ColN-WT and K145A mutant respectively. Superimposition of models created with BUNCH and *ab initio* methods demonstrated the additional volume in solution occupied by the disordered N-terminal fragment pointing towards conformational heterogeneity in solution. Sedimentation coefficients were calculated for these constructs (Table S1) and found to be in better agreement with measured values from sedimentation velocity experiments.

Nevertheless, the calculated BUNCH models correspond to unique, individual conformers whereby each of them fits the experimental scattering curve. This is in contrast to an anticipated mixture (ensemble) of structures in solution fitting the experimental curve as a whole. Consequently we applied the Ensemble Optimization Method (EOM) (9,10), which is able to deal with disordered polypeptide segments on their own or as part of a globular protein structure.

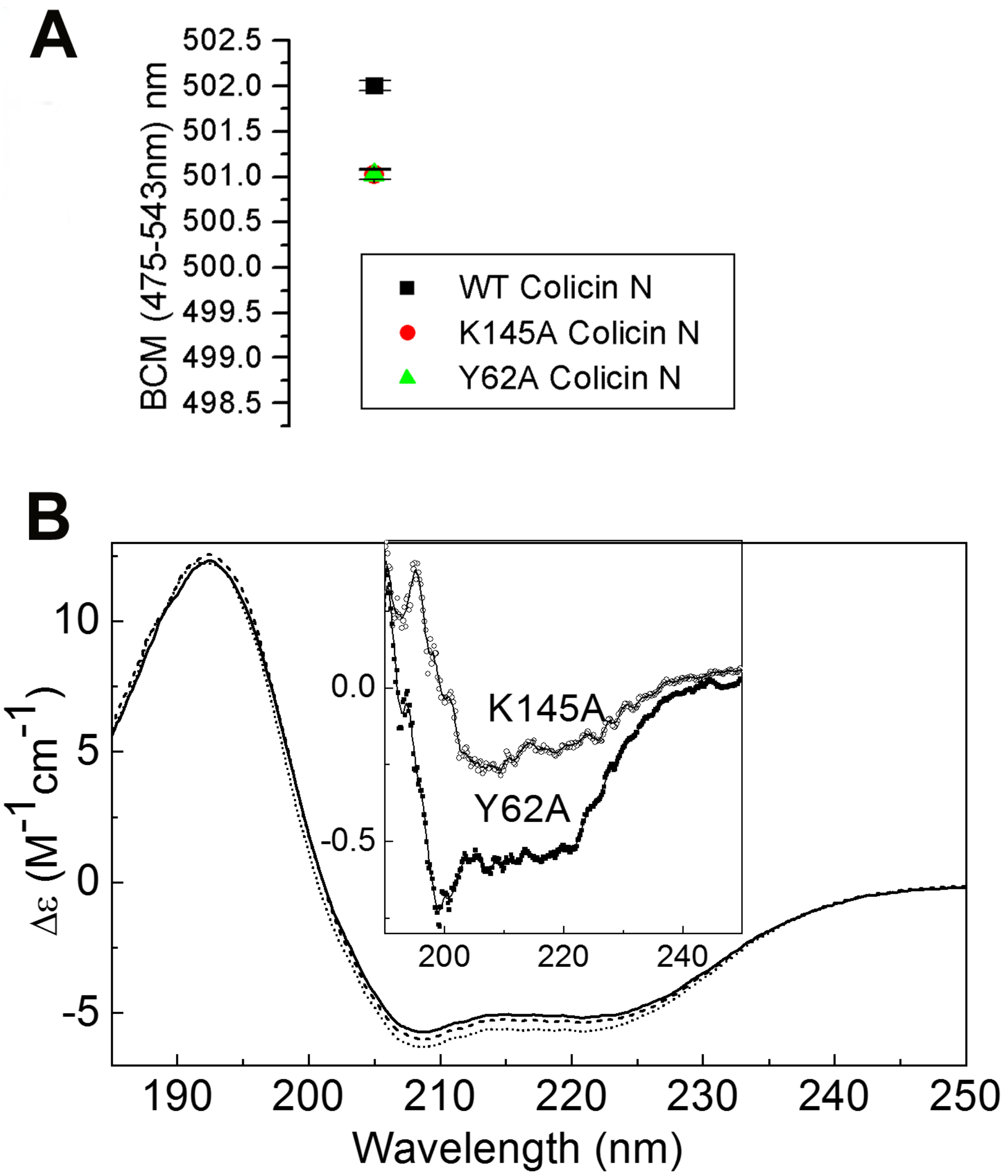

Figure S1. A) ANS binding reported by barycentric mean wavelength analysis (11). Both mutants show a 1 nm blue shift compared to WT indicating increased ANS binding. **B)** Far UV circular dichroism spectra of WT Colicin N (solid line), K145A mutant (dashed line) and Y62A mutant (dotted line). ***Inset:*** ColN-K145A and ColN-Y62A- minus WT difference spectra showing the detail of the increased signal in each.

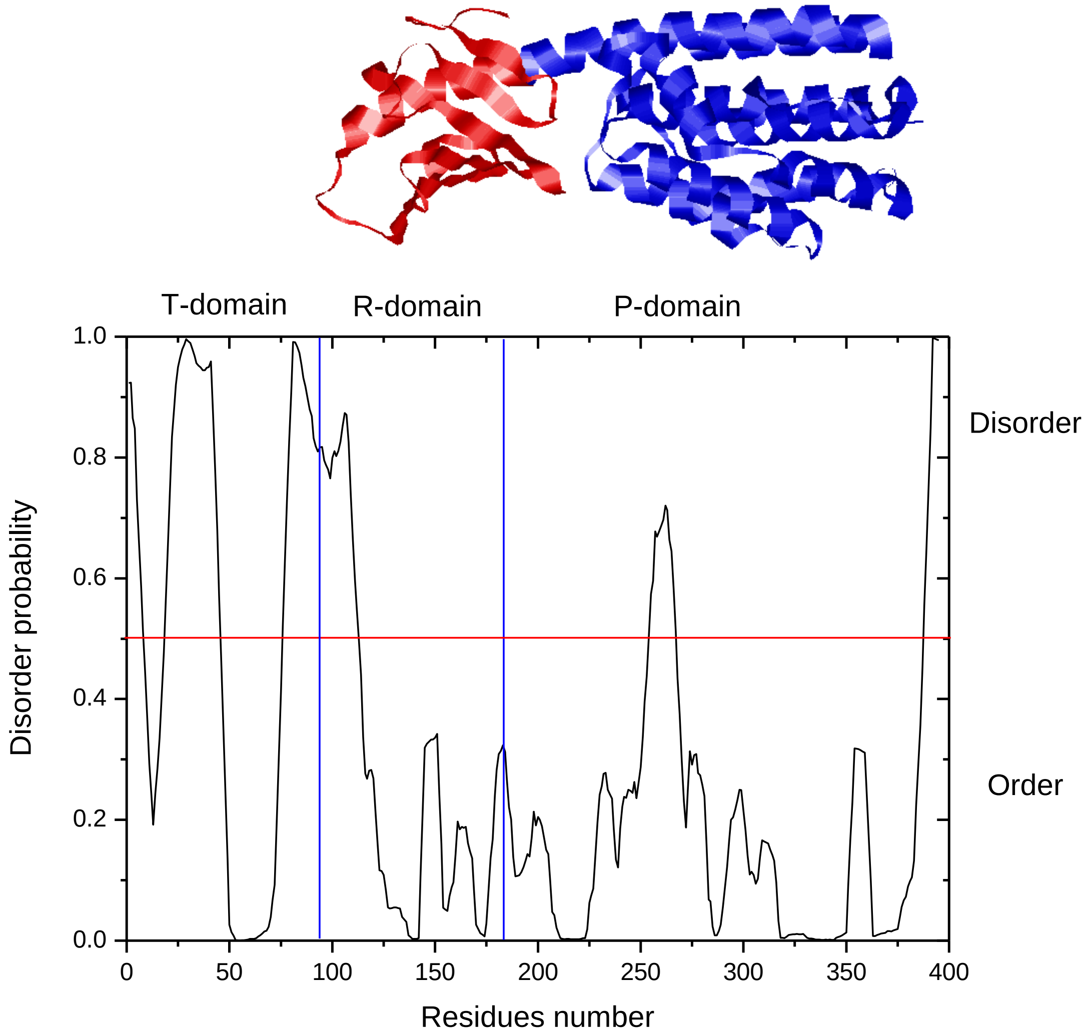

Figure S2. Structural disorder prediction for ColN full length molecule by the program PONDR (12). A high resolution structure of folded recognition (in red) and pore-forming (in blue) domains is shown approximately aligned with the prediction (pdb access code 1A87).

A

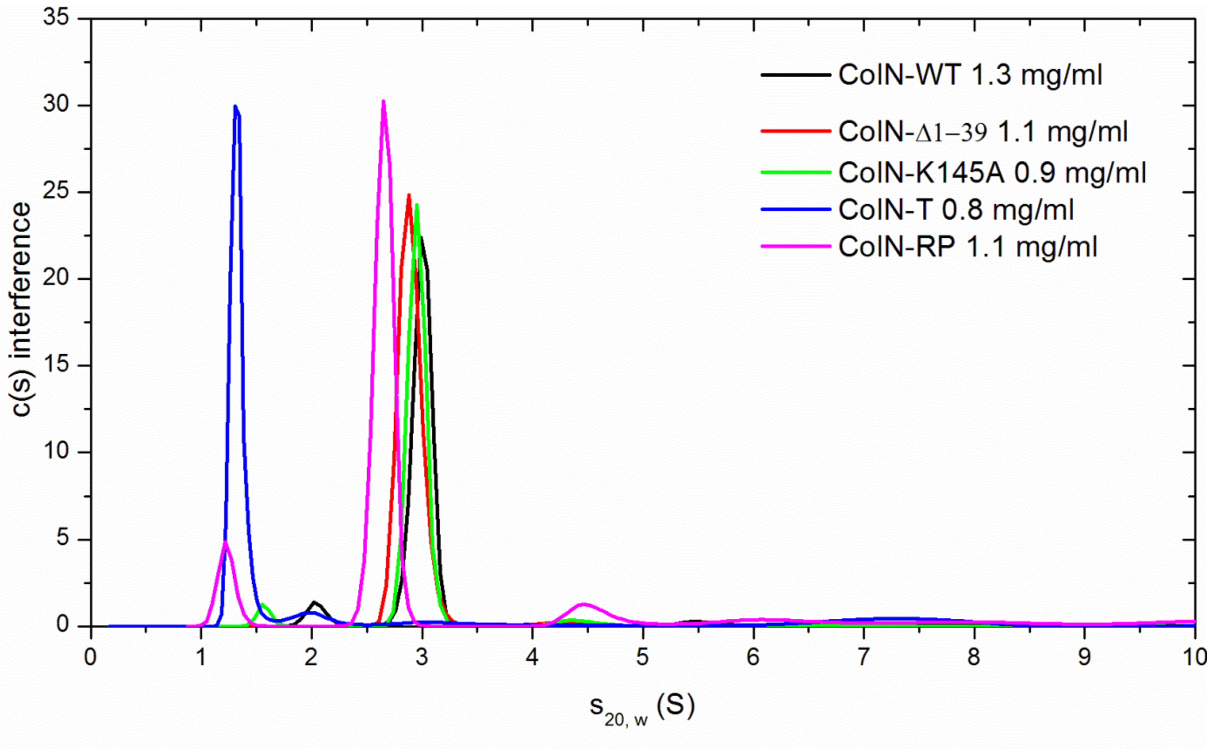

B

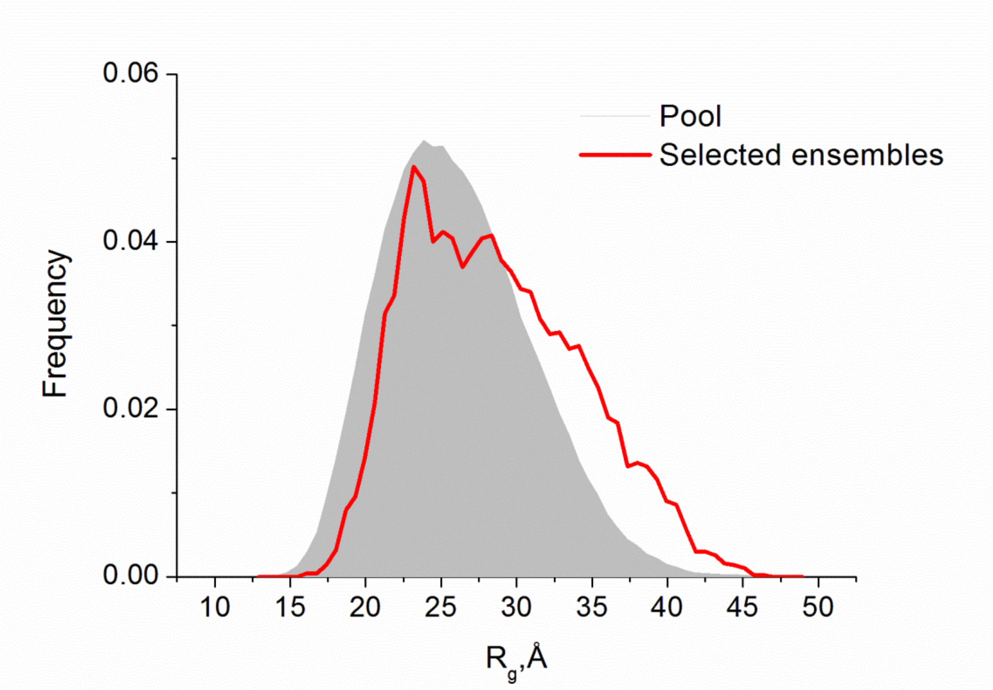

Figure S3.

A) All ColN constructs are virtually homogeneous monomers in solution.
Size distribution c(s) derived from sedimentation velocity boundaries. *Every sample is shown at a single concentration for clarity of representation.*

B) EOM modelled distribution of T -domain conformers in solution by their radii of gyration. Grey area represents general pool of structures. Red line represents the pool of selected ensembles.

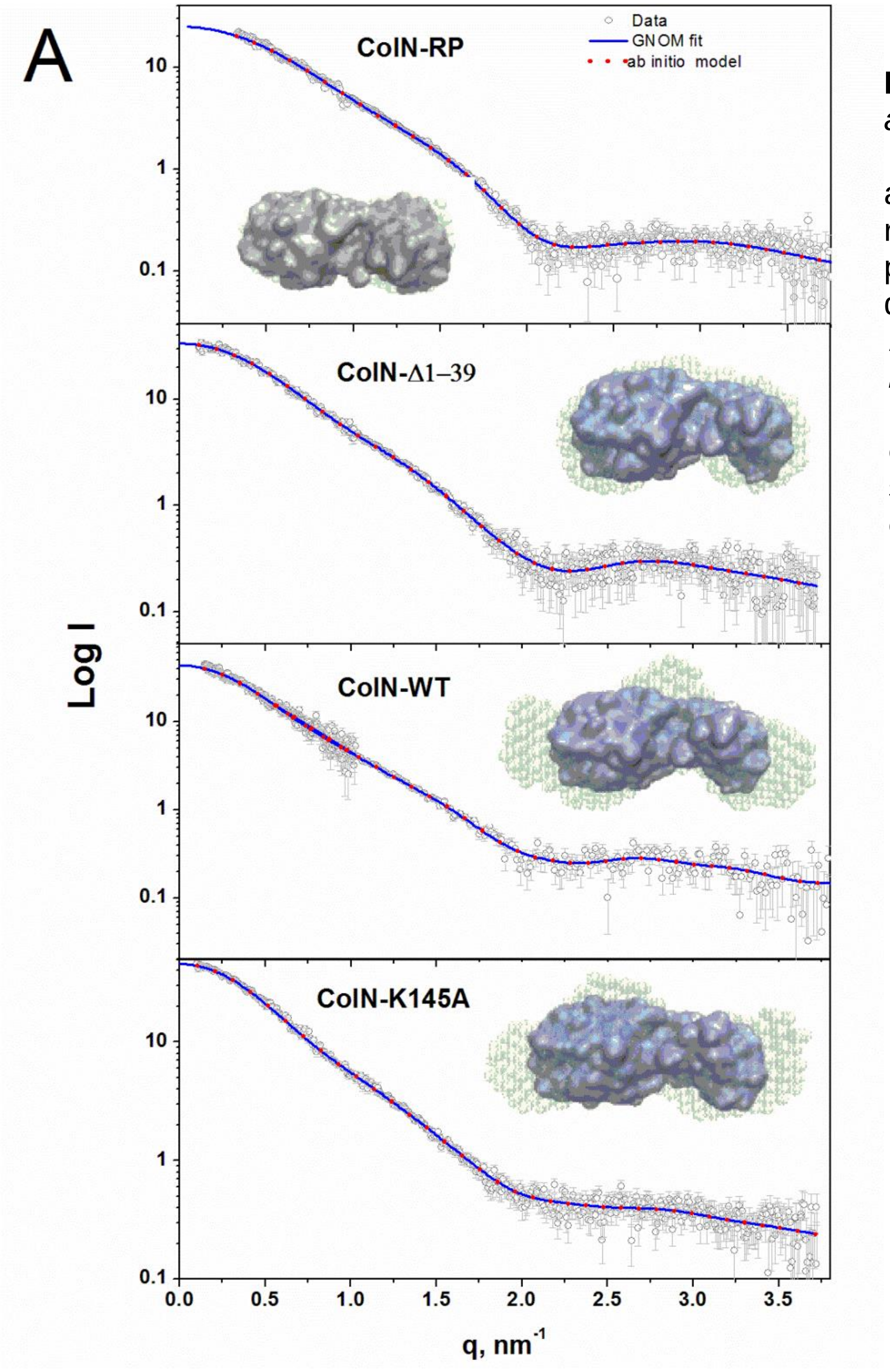

Figure S4. Rigid body modelling *ab-initio* based on SAXS data. Dummy Atom Models (DAMs) are superimposed upon the high-resolution structure comprising pore and receptor binding domains only (PDB 1A87).
A) Data fit and main view of *ab-initio* constructed DAM models
B) The model of ColN-WT created by the program BUNCH superimposed on the *ab-initio* constructed rigid body model.

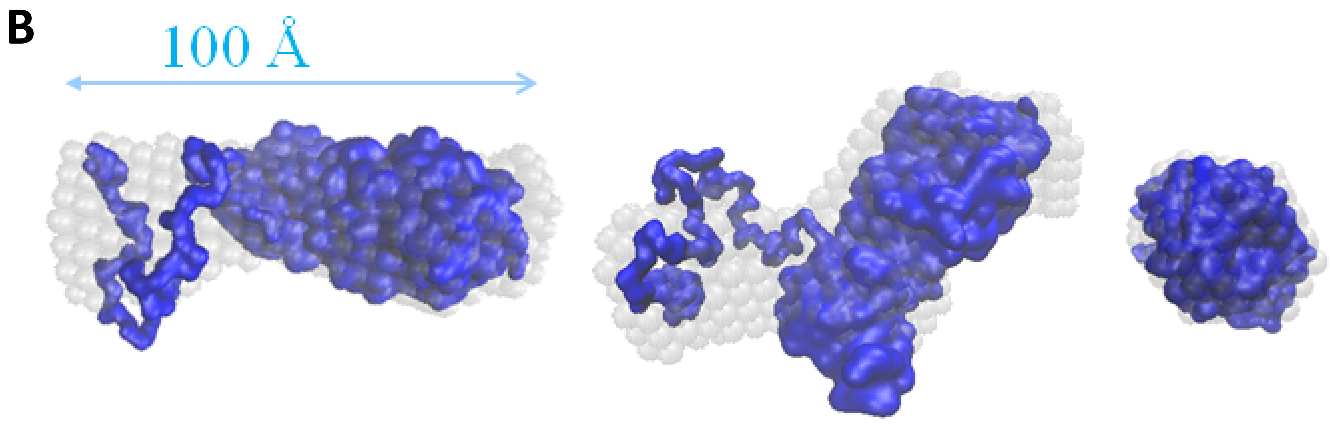

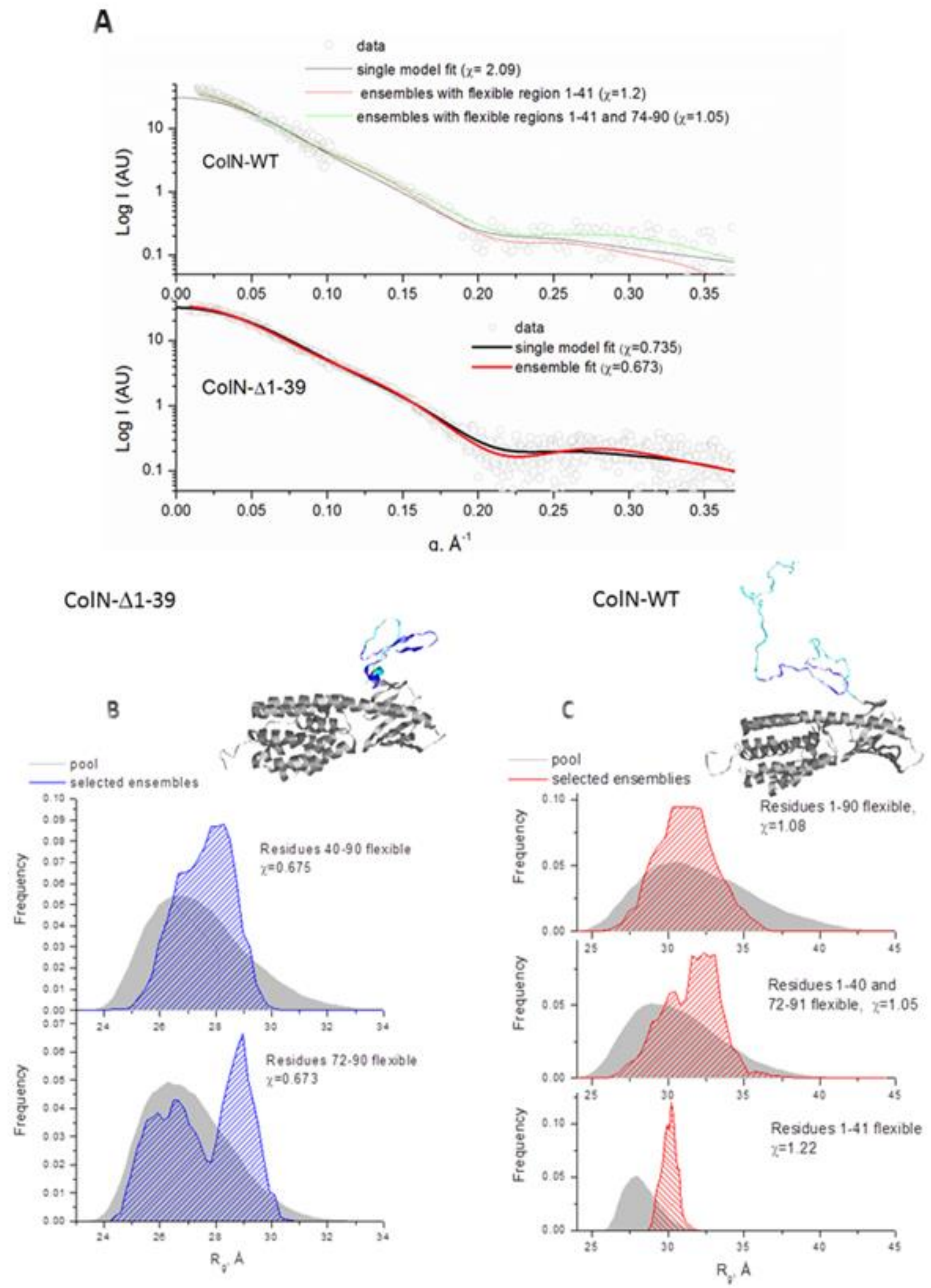

Figure S5.
A) CRYSOL (single model) and EOM data fit for iTasser generated models of ColN-WT and Δ1-39. (T-domain is shown in blue rigid TABS and cyan (flexible regions); **B)** and **C)** R_g distributions for iTasser generated ColN-Δ1-39 (B) and WT (C) structures with different degrees of flexibility.

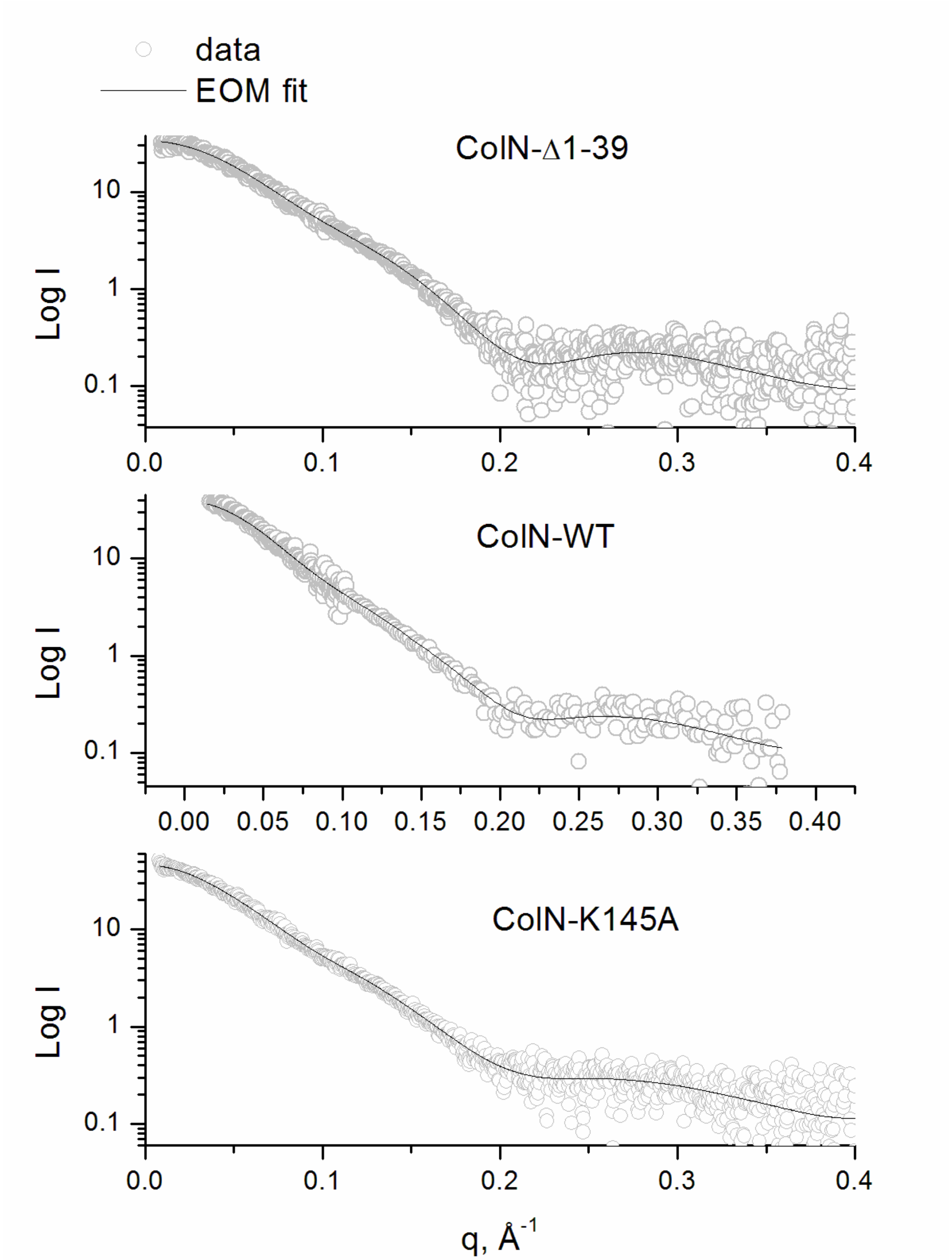

Figure S6. EOM fits to scattering data for ColN constructs, in each case T-domain was modelled as a completely flexible polypeptide using the program RANCH.

Supporting References

1. Schuck, P. 2000. Size-distribution analysis of macromolecules by sedimentation velocity ultracentrifugation and lamm equation modeling. Biophys. J. 78:1606-1619.
2. Vetter, I.R., Parker, M. W., Tucker, A. D., Lakey, J. H., Pattus, F., Tsernoglou, D. 1998. Crystal structure of a colicin N fragment suggests a model for toxicity. Structure 6: 863-874.
3. Svergun, D. I. 1992. Determination of regularisation parameter in direct-transform methods using perceptual criteria. J. Appl. Crystallog. 25:495-503.
4. Hansen, S. 2012. BayesApp: a web site for indirect transformation of small-angle scattering data. J.Appl. Crystallog.45:566-567.
5. Hansen, S. 2008. Simultaneous estimation of the form factor and structure factor for globular particles in small-angle scattering. J.Appl. Crystallog. 41:436-445.
6. Svergun, D. I. 1999. Restoring low resolution structure of biological macromolecules from solution scattering using simulated annealing. Biophys. J. 76:2879-2886.
7. Kozin, M. B., Svergun, D. I. 2001. Automated matching of high- and low-resolution structural models. J. Appl. Crystallog. 34:33-41.
8. Petoukhov, M. V., Svergun, D. I. 2005. Global rigid body modeling of macromolecular complexes against small-angle scattering data. Biophys. J. 89:1237-1250.
9. Bernado, P., Mylonas, E., Petoukhov, M. V., Blackledge, M., Svergun, D. I. 2007. Structural characterization of flexible proteins using small-angle X-ray scattering. J.Am.Chem. Soc. 129:5656-5664.
10. Bernado, P., Svergun, D. I. 2012. Structural analysis of intrinsically disordered proteins by small-angle X-ray scattering. Molecular BioSystems 8:151-167.
11. Chalton, D. A., Lakey, J. H. 2010 Simple Detection of Protein Soft Structure Changes. Analytical Chemistry 82:3073-3076.
12. Romero, P., Obradovic, Z., Li, X., Garner, E. C, Brown, C. J., Dunker, A. K. 2001. Sequence complexity of disordered protein. Proteins: Struct.Funct.Genet. 42: 38-48.

Table S1. Calculated sedimentation coefficient as a result of folding state compared with the result of AUC SV experiment

Sample	Calculated mass (kDa)	Calculated sedimentation hydrodynamic parameters										Experimentally determined (AUC)			
		Native state		Molten globule		Intrinsically disordered state				Denatured state[a]		s (S)[b]	M (kDa)[c]	R_s, Å	f/f_0^{shape}
						Premolten globule		Coil							
		R_s, Å	s, S	R_s, Å	s, S	R_s, Å	s, S	R_s, Å	s, S	R_s, Å	s, S				
ColN-T	9.965	16.73	1.44	19.16	1.26	23.57	1.02	26.31	0.92	28.07	0.89	1.33±0.045	9.88±0.291	20.60±1.45	1.36
ColN-RP	33.921	25.91	2.50	28.85	2.24	38.61	1.68	48.14	1.35	54.58	1.19	2.89±0.07	34.5±5.4	26.62±3.04	1.14
ColN-N40	39.212	27.28	2.84	30.28	2.56	40.94	1.89	51.71	1.50	59.05	1.40	2.84±0.003	38.8±0.81	31.97±1.95	1.26
ColN-WT	42.743	28.13	3.06	31.17	2.76	42.38	2.03	53.95	1.6	61.88	1.48	2.97±0.017	40.3±0.82	32.96±1.53	1.28
ColN K145A	41.685	28.12	3.06	31.15	2.76	42.36	2.03	42.36	1.6	61.83	1.48	2.94±0.007	41.75±1.22	34.50±2.50	1.33

[a]denatured by GdmHCl
[b]extrapolated to zero concentration
[c]averaged number

Table S2. SAXS results and parameters of the models constructed from SAXS curves

Sample	Parameters calculated from amino acid sequence		SAXS derived parameters				Hydrodynamic calculations (HYDROPRO)								Hydrodynamic calculations (SOMO)	
			GNOM		DAMMIN		Averaged (30 models) Dummy Atom Models (DAMs) built *ab-initio*				Rigid body models built using atomic coordinates (1A87) with added flexible chains (BUNCH)				Rigid body models built using atomic coordinates (1A87) with added flexible chains (BUNCH)	
	Mass. kDa	V_{anhydr}, nm^3	R_g, nm	D_{max}, nm	V_{DAM}, nm^3	$\delta\ g_w/g_{prot}$	AER	V_{hydr}, nm^3	s, S	$\delta\ g_w/g_{prot}$	AER, Å	V_{hydr}, nm^3	s, S	$\delta\ g_w/g_{prot}$	s, S	f/f_0
1A87*	35.15	43.5	–	–	–	–	–	–	–	–	3.5	61.61	2.802	0.31	2.81	1.21
ColN-RP	33.9	42.1	2.45	7.5	56.28	0.25	2.6	63.52	2.734	0.379	–	–	–	–	–	–
ColN-Δ1-39	39.2	48.2	2.75	8.5	60.52	0.19	3.0	75.84	2.953	0.425	3.3	66.46	2.836	0.281	2.97	1.35
ColN-WT	42.7	52.1	3.38	10.7	90.00	0.53	3.0	92.32	2.937	0.567	3.3	71.71	2.968	0.277	3.09	1.40
ColN K145A	41.7	52.0	3.15	10.3	77.26	0.36	3.3	91.18	2.981	0.553	3.8	78.76	2.982	0.377	3.18	1.37

*The hydrodynamic calculations from RP high-resolution structure are shown for comparison
